# Application of cold atmospheric microwave plasma on four domestic pigeons (*Columba livia domestica*) with open wounds

**DOI:** 10.17221/13/2025-VETMED

**Published:** 2025-06-27

**Authors:** Karla Zelaya, Jang-Hee Han, Jinyoung Kim, Seung Yoon Ahn, Young Deok Suh, Do Na Lee, Seong-Chan Yeon

**Affiliations:** Department of Veterinary Clinical Sciences, College of Veterinary Medicine and Research Institute for Veterinary Science, Seoul National University, Seoul, Republic of Korea

**Keywords:** avian, plasma medicine, wildlife, wound healing

## Abstract

This paper reports the feasibility of cold atmospheric microwave plasma (CAMP) therapy for birds with open wounds. Four domestic pigeons (*Columba livia domestica*) with open wounds of varying severity were treated with CAMP as an adjunct to conventional therapy. Wound healing was assessed using a modified scoring system during each treatment session, and the extent of healing was calculated as a percentage. The results revealed variability in wound improvement across cases, influenced by the initial severity of the wounds. In some cases, the outcomes were limited due to underlying conditions that overrode the recovery process, suggesting that the extent of healing may depend more on the initial wound condition than the treatment itself. Nevertheless, other cases showed positive results in healing and recovery under CAMP therapy, highlighting its potential benefits. No adverse reactions or complications were encountered, supporting its safety for avian wound management. These findings suggest that although CAMP can potentially promote wound healing, further studies will be needed to establish standardised treatment protocols.

Avian species comprise a huge portion of casualties admitted to wildlife rescue centres worldwide, with traumatic injuries being one of the most prevalent causes (Janssen et al. 2020). Constant threats, such as collisions, predator attacks, poaching, and environmental hazards, frequently result in open wounds, posing significant challenges to avian conservation efforts (Loss et al. 2012). In addition, the delicate anatomical structures of birds, including their thin skin layers and brittle bones prone to sharp fractures, increase the risk of open wounds (Bennett and Kuzma 1992; Sanchez-Ortiz et al. 2024). Managing these wounds in free-living species is challenging because of the limited opportunities for immediate intervention, such as primary closure (Burke et al. 2002). As a result, the wounds remain vulnerable to infections from various sources, including environmental pollutants, myiasis, predator-inflicted injuries, and even the bird’s plumage and skin microbiota (Benskin et al. 2009; Kim et al. 2023).

Although conventional wound care, such as topical antibiotics, bandaging, and surgical debridement, remains standard in veterinary practice, clinicians often encounter challenges in rescuing wildlife. Frequent bandage changes and prolonged antibiotic use can have adverse effects, including contact dermatitis, systemic toxicity, and disruption of tissue regeneration (Burke et al. 2002). Furthermore, the acquisition of multidrug-resistant bacteria in wild bird populations has been reported globally, which complicates wound conditions and ultimately affects post-release survival (Ramey and Ahlstrom 2020; Sanchez-Ortiz et al. 2024). The need for novel approaches that mitigate infection risks while ensuring faster healing has increased as the limitations of conventional therapies become evident. Indeed, proper wound care is crucial for promoting individual recovery and supporting population stability.

Plasma medicine is an emerging technology recognised for its potential in wound healing, infection control, and dermatologic disease care (Jung et al. 2023). Plasma, an ionised gas composed of reactive particles, is generated by applying energy such as electricity, heat, or microwaves to gases like argon (Jin et al. 2021). This process generates reactive oxygen and nitrogen species that eliminate pathogens, reduce inflammation, and promote cellular repair (Tan et al. 2022). The high temperature of plasma limits its direct use on living tissues. Hence, cold atmospheric microwave plasma (CAMP) was developed using continuous microwave energy to produce plasma at lower temperatures (Lertpatipanpong et al. 2023). This minimises the risk of thermal injury while preserving the antimicrobial and wound-healing benefits, making CAMP a valuable modality. The ability of CAMP to elicit strong biological responses at reduced temperatures makes it suitable for diverse species with varying tissue sensitivities, as demonstrated in dogs, cats, and mice, which exhibit accelerated wound closure (Jung et al. 2023; Yoo et al. 2023). In addition, *in* *vitro* studies have reported the efficacy of CAMP against a broad spectrum of microorganisms, including methicillin-resistant *Staphylococcus species*, which can cause significant skin issues in animals, such as birds (Briscoe et al. 2008; Jin et al. 2021).

Despite its growing success in mammals, the effect of CAMP in birds is unclear. The clinical significance of CAMP in avian medicine was evaluated by assessing the safety and effectiveness of CAMP in four domestic pigeons (*Columba livia domestica*) with open wounds and exploring its potential applications.

## Case presentation

### PATIENT SELECTION AND WOUND ASSESSMENT

Four domestic pigeons with different types of open wounds (abrasion, pressure sore, laceration, and gunshot) rescued by Seoul Wildlife Center were treated with CAMP as an adjunct to conventional treatment.

During each treatment session, macroscopic aspects of the wounds were evaluated using a modified wound scoring system adapted from Bates–Jensen Wound Assessment Tool (Harris et al. 2010) and Yoo et al. (2023) (Table 1). The findings were recorded using a customised wound grading chart (Table 2). Each parameter was scored on a scale from 0 to 3 (0 – none, 1 – mild, 2 – moderate, and 3 – severe), with total scores ranging from 0 to 24.

**Table 1:** Modified wound scoring system for evaluating the macroscopic parameters of the wound

Parameter	Score			
	0	1	2	3
Presence of foreign material	none	scant	moderate	excessive
Erythema	none	pink	bright red	dark red
Oedema	none	mild	moderate	severe
Papule	none	mild	moderate	severe
Exudate	none	scant	moderate	excessive
Debilitated tissue	none	mild	moderate	severe
Necrotic tissue	non-visible	white or grey	yellow slough	black eschar
Crust	none	mild thickness	moderate	severe

**Table 2:** Customised wound grading chart format used for recording the findings

ID number		Species		Entry date	
Wound location		Wound type		Initial weight	

Date	Session #	Wound size	Foreign material	Erythema	Oedema	Papule	Exudate	Debilitated tissue	Necrotic tissue	Crust	Total
											
											
											
											
											
											
											
											
											

Grade each existing lesion type: None – 0, Mild – 1, Moderate – 2, Severe – 3

The wound dimensions were gauged using a standardised ruler. The wound healing percentage was calculated at the end of the treatment period using the following formula:

$$\begin{array}{l} \text{Wound healing percentage}=[(\text{Initial wound}\\ \text{ score}-\text{Final wound score})\text{/Initial wound}\\ \text{ score}]\;\times 100 \end{array}$$ (1)

Initial wound score: total score recorded on day 1 of treatment; final wound score: total score recorded on the final day of treatment.

The same veterinarian performed the wound assessment, with a second veterinarian confirming the scores. Each wound was assessed individually if there were multiple wounds. All birds were monitored carefully for any signs of discomfort throughout the procedures, and all adverse reactions were documented.

### TREATMENT PROCEDURE

**Figure 1:**
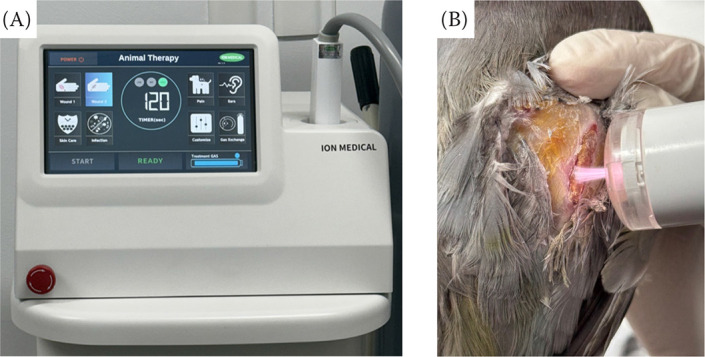
Cold atmospheric microwave plasma (CAMP) used for treating the open wounds of four domestic pigeons (*Columba livia domestica*) (A) CAMP device programmed to Wound 2 mode; (B) Application of CAMP on the wound of a domestic pigeon

The patients received conventional therapy as the baseline, with CAMP therapy applied only in the treatment group. The conventional therapy involved wound lavage and disinfection using a 0.5% chlorhexidine solution, followed by application of hydrogel (Duoderm Hydroactive Gel; ConvaTec, Seoul, Republic of Korea) and bandaging adjusted to the wounds whenever feasible. In the pigeons, CAMP therapy was carried out after wound disinfection using a plasma device (Bio Stimulation Microwave Plasma v1.0; IonMedical Inc., Seongnam, Republic of Korea) applied for 120 s, with a setting programmed to Wound 2 mode: an argon gas flow rate of 15 l/min, microwave frequency of 2.45 GHz, voltage of 3.5 kV, and power of 30 W (Figure 1).

The CAMP procedures were administered daily on weekdays following the routine protocols by Seoul Wildlife Center, after which the conventional protocol was resumed. All procedures were conducted with manual restraint and without sedation or anaesthesia. The treatment was discontinued upon complete wound healing, readiness for release, or euthanasia due to a poor prognosis. No complications related to the CAMP therapy were observed in any of the patients.

## Case summary

### Case 1

**Figure 2:**
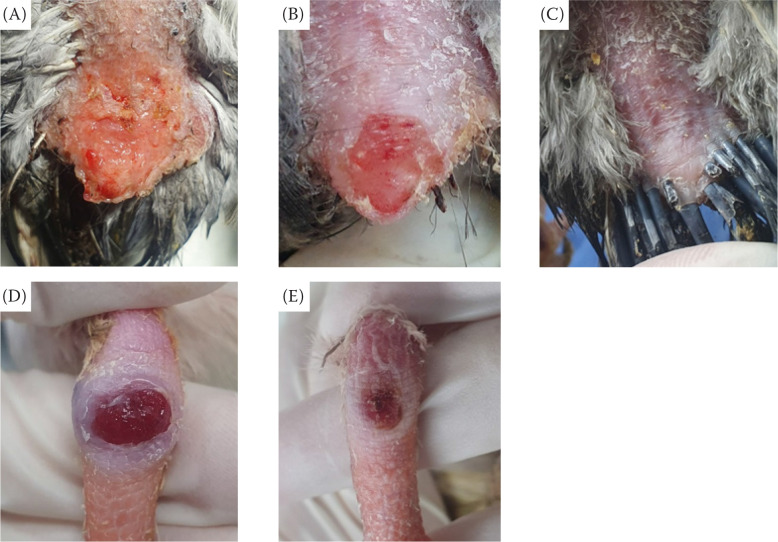
CAMP-treated open wounds in P1 (A–C) and P2 (D–E) In P1, the dorsal tail wound showed progression from the first day of treatment (A) to six days of treatment (B), with complete healing and feather regeneration observed after 18 days (C). In P2, the pressure sore on the left hock joint showed improvement from the first day of treatment (D) to significant healing after six days (E)

A domestic pigeon (P1) presented with a skin abrasion on the dorsal tail area from a predator attack. The initial wound area was 84 mm^2^ with a score of 5 and was unsuitable for bandaging. CAMP therapy was administered once daily for 11 consecutive days, totalling 11 sessions. The wound showed complete healing, with the wound area reduced to 0 mm^2^ and the score reaching 0. In addition, new feathers began to cover the affected area, showing remarkable improvement in healing (Figure 2A–C).

### Case 2

A domestic pigeon (P2) had a pressure sore on the left hock joint caused by ataxia. After seven sessions of CAMP therapy over seven days, the wound score decreased from 8 to 2, showing 75% improvement. The wound area was also reduced from 54.9 mm^2^ to 17.48 mm^2^ (Figure 2D,E). With significant wound recovery and overall condition improvement, the clinician discontinued the CAMP and moved the bird to a flight cage before release.

### Case 3

**Figure 3:**
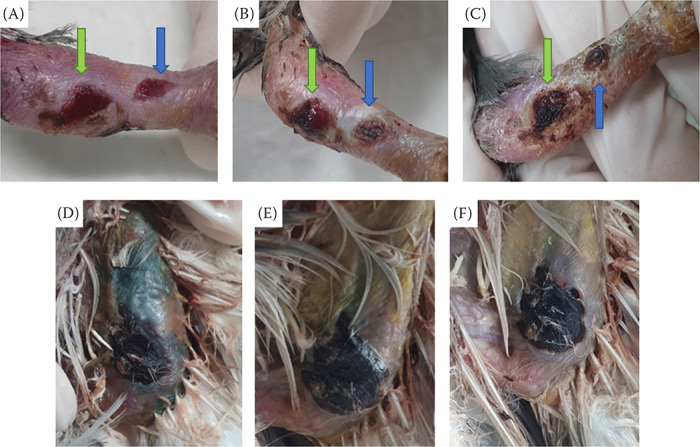
CAMP-treated open wounds in P3 (A–C) and P4 (D–F) In P3, two lacerations (lesions 1 and 2, indicated by green and blue arrows, respectively) on the tibiotarsus are shown. The images represent the first day of treatment (A), after three days (B), and four days (C). Despite partial wound healing, the limb was amputated due to a lack of nerve recovery. In P4, a gunshot wound on the forearm is depicted on the first day (D), after three days (E), and six days (F). While the wound also showed partial improvement, the CAMP was discontinued due to irreversible muscle damage, and the bird was euthanised

An orphaned pigeon (P3) presented with ataxia and two lacerations (lesions 1 and 2) on the left medial tibiotarsus. The affected limb showed signs of numbness, indicating potential nerve damage. Initial wound scores were 10 for both lesions, with lesion 1 measuring 19.8 mm^2^ and lesion 2 measuring 15.6 mm^2^. During a 3-day course of the CAMP therapy consisting of three sessions, the nerve function did not improve, necessitating limb amputation. Nevertheless, the partial wound healing was validated because lesion 1 showed 20% healing with a final score of 8 and an area increase to 21.28 mm^2^ due to granulation tissue formation. In contrast, lesion 2 showed 30% improvement with a final score of 7 and a reduced area of 14.08 mm^2^ (Figure 3A–C).

### Case 4

A domestic pigeon (P4) was referred with a gunshot wound on the left forearm with severe muscle damage. Radiography revealed a fractured ulna with an embedded BB bullet. Once surgical correction was performed, the initial wound score was 12, with an area measuring 142 mm^2^. After six CAMP sessions conducted over six days, the wound score was reduced to 9, and the area decreased to 121 mm^2^, indicating a 25% improvement (Figure 3D–F). The treatment was stopped because of irreversible muscle damage with a severely compromised limb function, and euthanasia was performed.

Table 3 lists the demographic profiles and treatment summaries for all birds.

**Table 3:** Demographic profiles and treatment results of four domestic pigeons (*Columba livia domestica*)

Patient ID	Species	Wound type	Wound location	Cause of admission	Treatment type	Baseline wound area (score)	Final wound area (score)	Total sessions	Improvement
P1	Domestic pigeon	Skin abrasion	dorsal tail area	Predator attack	CAMP	84 mm^2^ (5)	0 mm^2^ (0)	11	100%
P2	Domestic pigeon	Pressure sore	left hock joint	collision	CAMP	54.9 mm^2^ (8)	17.48 mm^2^ (2)	7	75%
P3	Domestic pigeon	two skin laceration	left medial tibiotarsal area	orphan, left leg trauma	CAMP	lesion 1: 19.8 mm^2^ (10) lesion 2: 15.6 mm^2^ (10)	lesion 1: 21.28 mm^2^ (8) lesion 2: 14.08 mm^2^ (7)	3	lesion 1: 20% lesion 2: 30%
P4	Domestic pigeon	Gunshot wound	left forearm	predator attack	CAMP	142 mm^2^ (12)	121 mm^2^ (9)	6	25%

CAMP = cold atmospheric microwave plasma

## DISCUSSION

After reviewing the progress of the four cases, the CAMP therapy exhibited potential for promoting open wound healing in certain cases (P1 and P2), but it was not consistently applicable in others (P3 and P4). In P1, the wound fully healed after 11 days of CAMP therapy, highlighting its practicality in areas unsuitable for bandaging or sutures. Comparable benefits were also observed in companion animals, such as dogs and cats, particularly in wounds in anatomically challenging areas such as paws, joints, and other high-risk regions (Yoo et al. 2023). In addition, P2 showed a 75% improvement within seven days, further supporting the early effectiveness of CAMP in appropriate cases. Research on murine models corroborated these results, showing accelerated wound closure, improved epithelialisation, and strengthened tissue (Schmidt et al. 2017). CAMP therapy could offer advantages in wild bird rehabilitation by reducing the captivity duration because birds also follow a similar cutaneous healing mechanism to mammals (Burke et al. 2002). Moreover, its non-thermal nature minimises irritation, a crucial factor in avian medicine where stress-prone properties and their behaviours make long-term bandaging challenging (Ferrell 2002; DuRant et al. 2016).

In contrast, P3 and P4 displayed the challenges of applying the CAMP therapy under unfavourable conditions. P3 shows two skin lacerations with limb dysfunction, requiring amputation because of the irreversible nerve damage unrelated to the wound itself. Although partial wound healing was observed, the neurological complications overshadowed the localised benefits of the therapy. Similarly, P4 suffered a severe gunshot wound with extensive tissue loss, ultimately leading to euthanasia despite modest improvements from CAMP therapy. These cases highlight the limited efficacy of the treatment in severe trauma where the structural integrity and tissue viability are compromised. Therefore, although CAMP can support localised healing, it remains an adjunctive treatment, requiring systemic management to address severe trauma or infection. Nevertheless, localised wound healing rates of 20–30% and 25% in P3 and P4, respectively, highlight its potential utility even under challenging conditions. Additionally, the comparable per-session healing rate in P3 to that of P1 and P2 further supports its effectiveness.

Interestingly, no adverse effects were observed during treatment, and the regrowth of feathers at the healed sites was a remarkable outcome. Hence, repeated exposure to the CAMP is safe for avian species, potentially preserving feather follicles and enhancing wound management. In addition, transient erythema and mild discomfort, evidenced by vocalisation or movement, were observed in CAMP-treated dogs, but such troubles were absent in avian subjects (Yoo et al. 2023). Despite the inherent limitations in evaluating the adverse effects in birds due to the masking phenomenon, the absence of observable complications reinforces the safety and applicability of CAMP therapy in avian wound care.

The anti-infective properties of CAMP enhance its potential as an alternative solution for managing contaminated wounds, particularly in multidrug-resistant (MDR) bacteria, which have also been detected in the scars of wild birds (Tardon et al. 2021; Sanchez-Ortiz et al. 2024). A major challenge in treating MDR-infected wounds lies in the biofilms produced by the pathogens. Biofilms are composed of polysaccharides, lipids, proteins, and nucleic acids that form a protective matrix, enhancing bacterial resilience to antibiotics and environmental stresses, significantly complicating treatment (Olsen 2015). In this context, approaches using cold plasma offer a novel alternative by disrupting biofilms and restoring bacterial susceptibility to antibiotics, thereby addressing one of the major barriers to treating MDR infections (Bayliss et al. 2013; Brun et al. 2018). The following studies indicate that plasma can achieve significant bacterial eradication within minutes of application, underscoring its potential to combat biofilm-associated antimicrobial resistance effectively (Jin et al. 2021; Kim et al. 2022). Considering the increasing threat of MDR bacteria, CAMP therapy offers a dual benefit: promoting localised wound healing while addressing broader infection risks. Its ability to disrupt biofilms and reduce antibiotic resistance underscores its potential as a critical component of modern wound management strategies in avian medicine.

This study had several limitations. The small sample size restricted the generalisability, and the absence of microbiological cultures hindered an evaluation of the antimicrobial effects of CAMP. The use of a single operational mode (Wound 2) also limited the comparisons between treatment protocols. Additionally, the potential for applying CAMP to other types of avian wounds, such as burns, frostbite, or ligature wounds, is yet to be explored. Nevertheless, the study highlights CAMP therapy as a promising and safe treatment for avian wounds. Moreover, it offers a viable alternative for addressing the growing concern of multidrug-resistant bacterial infections. Future research with larger cohorts, varied injury types, and microbiological analyses will be needed to validate these findings and broaden the use of CAMP in avian wound care, particularly in wildlife rehabilitation and conservation.
